# Adult Neurogenesis Is Altered by Circadian Phase Shifts and the Duper Mutation in Female Syrian Hamsters

**DOI:** 10.1523/ENEURO.0359-22.2023

**Published:** 2023-03-28

**Authors:** Michael Seifu Bahiru, Eric L. Bittman

**Affiliations:** 1 Program in Neuroscience and Behavior, University of Massachusetts, Amherst, Massachusetts 01003; 2 Department of Biology, University of Massachusetts, Amherst, Massachusetts 01003

**Keywords:** circadian rhythms, Cry1, cryptochrome, adult neurogenesis, duper mutation, entrainment, jet lag

## Abstract

Cell birth and survival in the adult hippocampus are regulated by a circadian clock. Rotating shift work and jet lag disrupt circadian rhythms and aggravate disease. Internal misalignment, a state in which abnormal phase relationships prevail between and within organs, is proposed to account for adverse effects of circadian disruption. This hypothesis has been difficult to test because phase shifts of the entraining cycle inevitably lead to transient desynchrony. Thus, it remains possible that phase shifts, regardless of internal desynchrony, account for adverse effects of circadian disruption and alter neurogenesis and cell fate. To address this question, we examined cell birth and differentiation in the duper Syrian hamster (*Mesocricetus auratus*), a *Cry1*-null mutant in which re-entrainment of locomotor rhythms is greatly accelerated. Adult females were subjected to alternating 8 h advances and delays at eight 16 d intervals. BrdU, a cell birth marker, was given midway through the experiment. Repeated phase shifts decreased the number of newborn non-neuronal cells in WT, but not in duper hamsters. The duper mutation increased the number of BrdU-IR cells that stained for NeuN, which marks neuronal differentiation. Immunocytochemical staining for proliferating cell nuclear antigen indicated no overall effect of genotype or repeated shifts on cell division rates after 131 days. Cell differentiation, assessed by doublecortin, was higher in duper hamsters but was not significantly altered by repeated phase shifts. Our results support the internal misalignment hypothesis and indicate that *Cry1* regulates cell differentiation. Phase shifts may determine neuronal stem cell survival and time course of differentiation after cell birth. Figure created with BioRender.

## Visual Abstract

**Figure:**
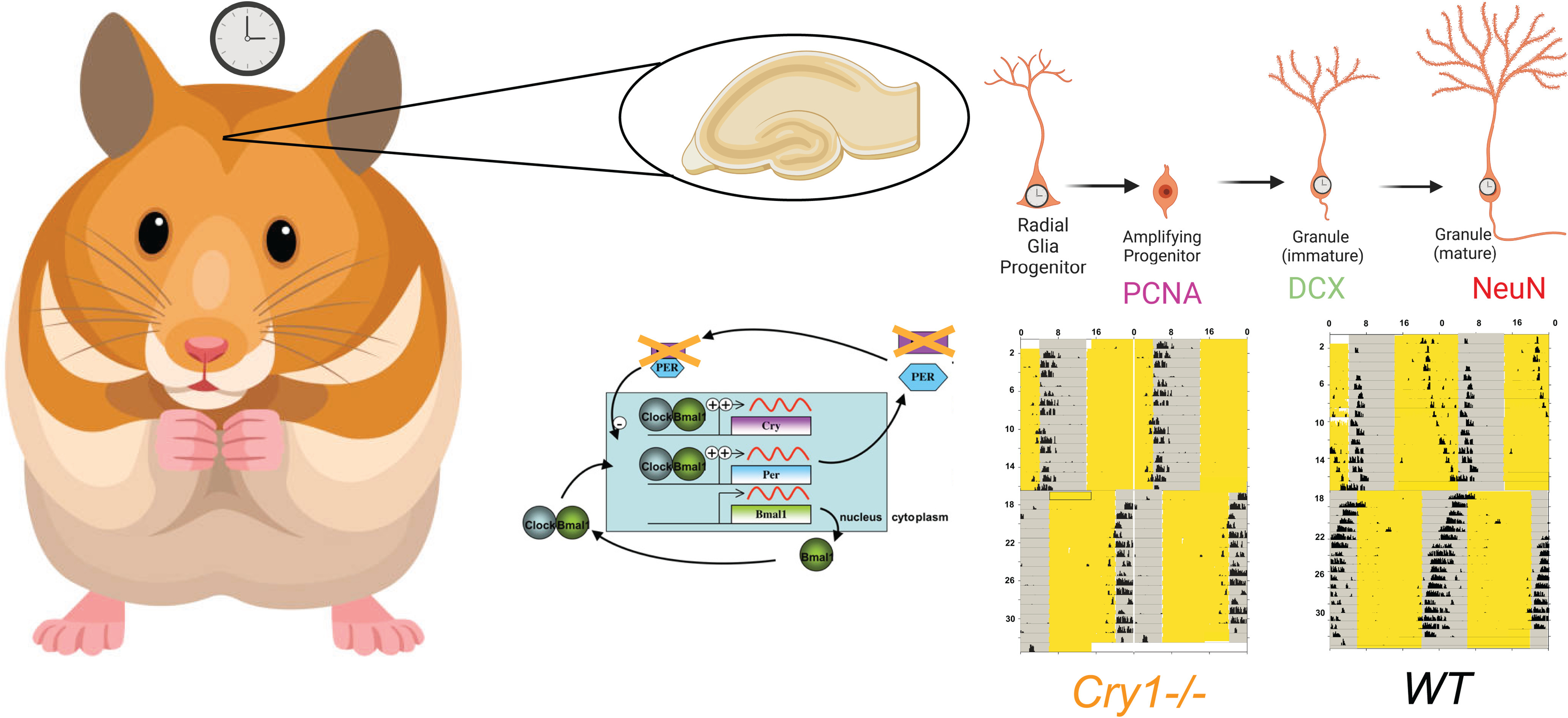

## Significance Statement

The birth of neurons in the adult brain impacts learning and memory. Circadian disruption, such as occurs in jet lag, adversely affects neurogenesis. It is unclear whether shifts of the light/dark cycle are inherently deleterious, or whether misalignment of the phase of circadian oscillators is responsible. Repeated shifts decreased the number of non-neuronal cells born in adult dentate gyrus of wild-type hamsters, but increased the percentage that developed neuronal phenotype. Duper mutants, which are deficient in the core clock gene *Cryptochrome 1* and re-entrain four times as fast as wild types, experienced increased neurogenesis but showed no effect of phase shifts. These results implicate *Cry1* in the regulation of neurogenesis and indicate that circadian misalignment is critical in jet lag.

## Introduction

Stem cells divide, and new neurons are generated in the subgranular zone (SGZ) of the dentate gyrus of the hippocampus of adult rodents (Gonçalves et al., 2016; Obernier and Alvarez-Buylla, 2019). Improvements in learning and memory are correlated with increases in adult-born neurons in the dentate gyrus (Gould et al., 1999). Conversely, memory deficits and psychological disorders may both influence and result from the disruption of adult neurogenesis (Jessberger et al., 2009; Moreno-Jiménez, et al., 2019; Terranova et al., 2019). The generation and survival of neurons born in adulthood may be regulated by environmental factors (Dranovsky et al., 2011; Toda et al., 2019). The disruption of circadian organization, such as occurs in rotating shift work, irregular sleep patterns, and jet lag, is correlated with numerous diseases and psychological disorders (Costa, 2010; Gibson et al., 2010; Logan et al., 2015). Chronically jet-lagged adult hamsters, rats, and mice show a decrease in hippocampal cell proliferation and maturation (Gibson et al., 2010; Kott et al., 2012; Iggena et al., 2017; Horsey et al., 2020). These findings have drawn attention to the extent and mechanisms of circadian regulation of neurogenesis in the adult hippocampus.

The circadian program serves not only to optimize temporal scheduling of internal events, but also to establish appropriate phasing with respect to the environment. The daily light/dark (LD) schedule provides the most powerful entraining cue (zeitgeber) to the circadian system. The molecular clocks found in plants, fungi, and animals rely on transcriptional translational feedback loops (TTFLs; for review, see Takahashi, 2017). In mammals, the protein products of *Brain and Muscle ARNT-Like 1* (*Bmal1*) and *Clock* or *Npas2* dimerize and act as transcriptional activators at E-box motifs in promoter regions of the *Cryptochrome* (*Cry 1*,*2*) and *Period* (*Per 1*,*2*,*3*) genes. After transcription, translation, dimerization, phosphorylation, and nuclear translocation, PER and CRY complete a negative feedback loop by inhibiting the action of BMAL1:CLOCK heterodimers. These clock genes form the core of a cell-autonomous circadian oscillator, as evidenced by changes in period or arrhythmicity on their mutation or knockout. Furthermore, they directly or indirectly regulate the rhythmic expression of thousands of other “clock-controlled” genes, which serve critical physiological functions in the brain and periphery (Koike et al., 2012). Circadian oscillations in the expression of core clock *Per1* and *Bmal1* regulate cell cycle entry and exit in the hippocampus of adult mice (Bouchard-Cannon et al., 2013; Harbour et al., 2014).

What accounts for the decrement in cognitive performance and neurogenesis in animals subjected to repeated shifts of circadian phase? The principal hypothesis is that circadian oscillators in different cell types, brain regions, and peripheral organs shift at different rates such that re-entrainment is a protracted process during which the phase of multiple oscillators is misaligned. Although supported by observations that the rate of phase shifts varies in different organs (Yamazaki et al., 2000; Davidson et al., 2009), this “internal desynchrony” hypothesis has been difficult to test. For example, changes of phase per se, independent of the duration of a misaligned state, could be deleterious to physiological function and cognitive performance (West et al., 2017).

Duper, a circadian mutant that dramatically accelerates the rate of re-entrainment in hamsters subjected to shifts of the LD cycle, can be a useful tool to investigate the role of circadian alignment in adult neurogenesis (Krug et al., 2011; Monecke et al., 2011; Sisson et al., 2022). A deletion of a cytosine in exon 4 of *Cry1* of this mutant causes a frameshift leading to a stop codon. As a result, duper hamsters lack detectable CRY1 in the liver and brain (Lee et al., 2022). We have taken advantage of the re-entrainment properties of duper hamsters to examine the impact of phase shifts and circadian misalignment on adult neurogenesis. If the adverse effects of phase shifts are due to the delay in realignment, neurogenesis will be less compromised in duper mutants than in wild types (WTs).

## Materials and Methods

### Maintenance of adult hamsters and photoperiod manipulation

Adult female Syrian Hamsters (LVG strain; bred from animals purchased from Lakeview Hamstery) were maintained in a 14:10 h L/D cycle from birth. The animals had *ad libitum* access to food and water. Beginning at ∼85 d of age, animals were single housed in a plastic tub cage with a metal running wheel (diameter, 17 cm). Locomotor activity was tracked throughout the experiment using ClockLab Actimetrics software (version 2.4.2). A total of 34 animals was used in the experiment. All procedures and methods were approved by the institutional animal care and use committee.

### Phase-shifting paradigm

**Figure 1.**
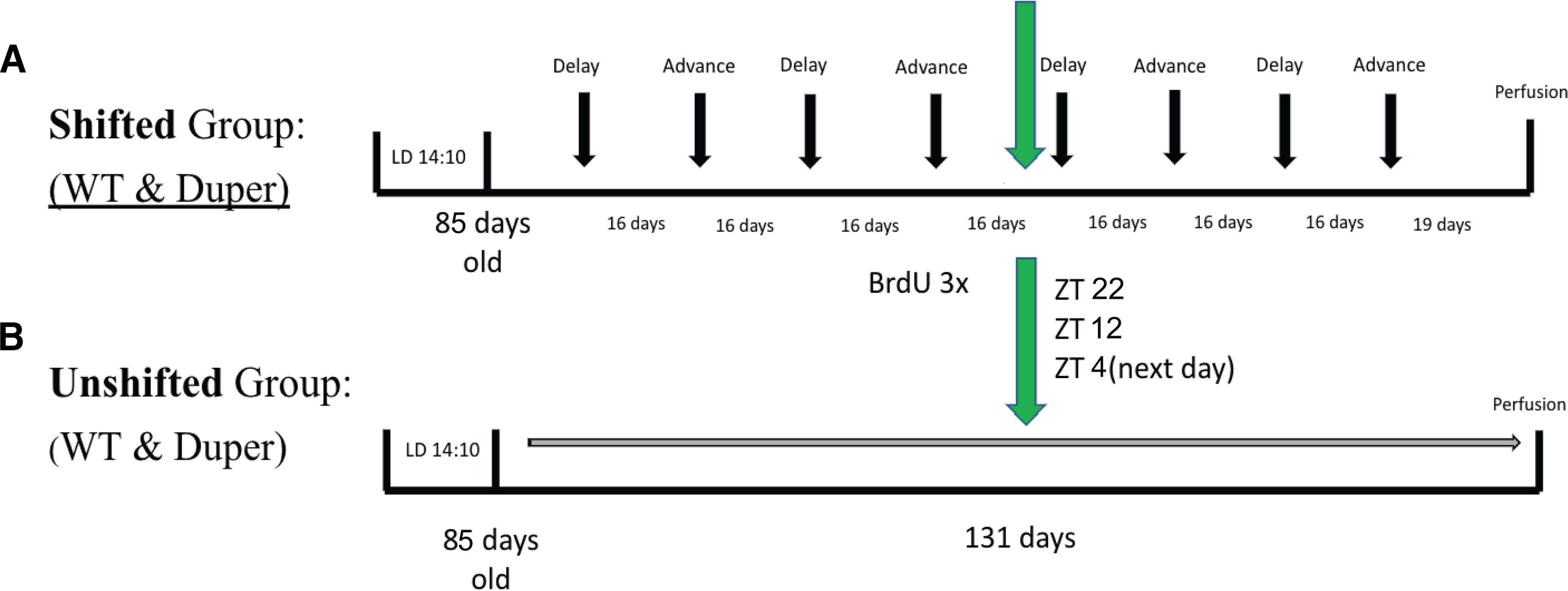
Experimental design. ***A***, The shifted group was composed of 8 wild-type and 8 duper female hamsters. ***B***, The unshifted animals also contained 8 of each genotype and served as controls. The green arrow indicates the time point of BrdU injections at the end of the second advance. We injected animals at ZT22 and ZT12 on the first day and ZT4 on the second day for both the shifted group and the control group. Four more phase shifts (2 delays and 2 advances) were administered after the BrdU injections. Hamsters were perfused 16 d after the last advance shift, after which brains were sectioned and double-label immunocytochemistry was performed to detect BrdU and NeuN, PCNA, and DCX proteins.

Animals were assigned to shifted and unshifted groups (*n* = 16/group). Each group was composed of WT (*n* = 8) and duper (*n* = 8) animals. The unshifted group remained in an unchanging 14:10 h L/D cycle (lights on 7:00 A.M.; Fig. 1). The shifted group was subjected to alternating 8 h phase delays (lights on at 3:00 P.M.) and advances beginning at ∼85 d of age. Shifts were repeated at 16 d intervals, so that hamsters experienced a total of eight shifts. Phase delays were accomplished by extending the light phase while phase advances were implemented by delaying the dark phase. Entrainment to the shifted LD cycle was determined to have occurred when the slope of a linear least-squares regression line fit to activity onsets paralleled the line fit to lights off on the actogram. Daily vaginal smears were conducted in the early light phase (ZT3) to track estrous cycle throughout the experiment. The first four shifts were done on proestrus, and subsequent shifts were done on metestrus.

### Cell division marker injections

At the end of the fourth shift (the second advance), animals were administered bromodeoxyuridine (BrdU; 50 mg/kg in 0.07N NaOH; Sigma-Aldrich). In light of evidence that cell division varies with the time of day (Smith et al., 2010), multiple injections were given at different zeitgeber times. The first injection was at lights-on (ZT22; 7:00 A.M.), the second at lights-off (ZT12; 9:00 P.M.), and the third and last injection was midday the following day [ZT6 (estrus); 1:00 P.M.]. To avoid possibly confounding the influences of ovarian steroids (Tanapat, et al., 2005), age-matched and estrus-matched days were used to inject animals in the unshifted group.

### Tissue collection

The animals were anesthetized with 0.1 ml of sodium pentobarbital (80 mg/kg) at ZT8. Transcardial perfusion with 100 ml of 0.1 m phosphate buffer (PB) was followed by 300 ml of 4% paraformaldehyde. Once the brain was extracted, it was postfixed in 4% paraformaldehyde for 15 h at 4°C before transfer to 20% sucrose in 0.1 m PB at 4°C. After 2 d of infiltration, the brains were sectioned at 40 μm using a freezing microtome. Tissues were collected in a 1 in 6 series and stored in cryoprotectant at −20°C until stained.

### Immunocytochemistry

To examine cell division in the two major germinal regions of the adult brain, four sections were taken from the subventricular zone (SVZ) in the wall of the lateral ventricle at the preoptic striatal level, and five sections from the hippocampal SGZ of each animal. These regions span most of the preoptic area for SVZ and most of the hippocampus for SGZ. All sections were stained simultaneously in a single run. To minimize tissue handling, 6 × 10 mm mesh-bottomed wells were used to wash the sections with PBS. The sections were washed 4× in PBT (PBS with Triton X-100) followed by a rinse in saline 0.9% for 10 min. Sections were denatured in 2N HCl for 30 min at 37°C, neutralized with 0.1 m sodium borate buffer, pH 8.5, for 30 min at room temperature, and rinsed 3× (5 min each) in PBT. Once the sections were washed in PBT 3×, they were blocked in PBS^+^ (PBT containing 0.1% bovine serum albumin) with 4% normal donkey serum for 1 h. Sections were then incubated in primary antibody overnight (14–18 h) on a rocker at room temperature.

Double-label immunocytochemistry was used to detect BrdU and NeuN protein. The primary antibodies were rat anti-BrdU (1:350; Accurate Chemical & Scientific Corporation), mouse anti-NeuN (1:1000; Chemicon), mouse anti-proliferating cell nuclear antigen (PCNA; 1:300; Santa Cruz Biotechnology), and rabbit anti-doublecortin (DCX; 1:100; Proteintech). Secondary antibodies were Cy3 donkey anti-rat, Cy5 donkey anti-mouse, and Cy3 donkey anti-rabbit (all used at 1:350; Jackson ImmunoResearch). All antibodies were diluted in PBS+ (0.1 m PBS containing 0.4% Triton X-100 and 0.1% BSA fraction V).

After washing (4× PBS) and incubation in secondary antibody for 2 h at room temperature, the sections were washed (2×) and placed in DAPI (1:1000) for 30 min at room temperature. The sections were washed in 0.1 m PB, mounted on subbed slides, and left to dry overnight. The sections were coverslipped (Aqua-Poly/Mount, Polysciences), set to dry for 2 d, and stored until imaging.

### Imaging, cell counting, and quantification

Images were collected in the form of *z*-stacks at 20× (10× objective) and 16 bit with a confocal microscope (model 710, Zeiss). The gain used for the first channel (Cy3) is 620 and a gain of 681 was used for the second channel (Cy5). Once the upper limit and lower limit of each section were established, regions of interest (ROIs) were identified spanning one section at a time. Once every ROI is established, *z*-stacks for every selected region start being collected for both channels automatically through the Series option in the Zeiss confocal software (Schindelin et al., 2012). Files are collected in the .czi format with FIJI (ImageJ; http://fiji.sc/Fiji) . NIH imaging software was used to convert files automatically in batch form with a macro developed in our laboratory. Once conversions were completed, a researcher blind to animal identification (genotype/condition) conducted manual cell counts and colabeling. MSB determined total BrdU cell counts as well as colabeled BrdU and NeuN cells. This was determined manually using MATLAB 2019A, which allowed us to separate the different channels and verify colabeling. The same was done to identify PCNA^+^ and DCX^+^ cells. For the latter, process length was determined using the draw feature of FIJI to manually draw lines over the projections within each stack and take the average length per image.

### Statistical analyses

All statistical analyses were performed using GraphPad Prism (version 8.4.2 for macOS; GraphPad Software; www.graphpad.com). Two-way ANOVAs using a mixed-effects model were used to evaluate the main effects of phase shifts and genotype, and their interaction for cell counts and colabeling. *Post hoc t* tests were used to compare shifted and control groups within the same genotype. Multiple *t* tests were used to analyze the latency of re-entrainment of activity rhythms.

## Results

### Re-entrainment of duper and WT

**Figure 2.**
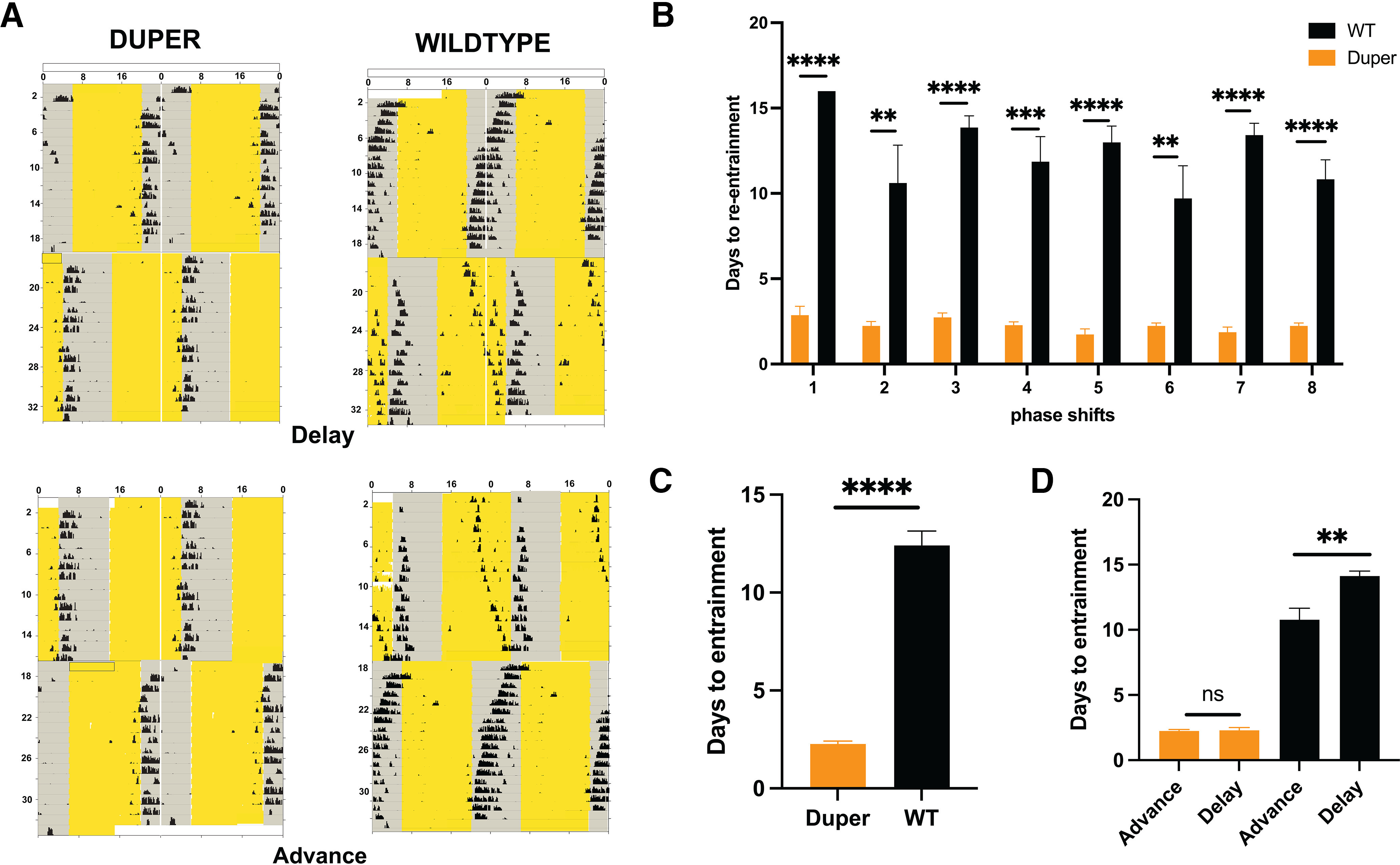
Duper hamsters re-entrain more rapidly than wild types to shifts of the LD cycle. ***A***, Double-plotted actograms of representative duper (left) and wild-type (right) hamsters subjected to successive 8 h phase delays (top) and advances (bottom) of the 14:10 h L/D cycle. Yellow shading indicates light phase. Mutants re-established the stable phase relationship of locomotor activity onset to the onset of the dark phase within 4 d, but WT hamsters required ∼14 d to re-entrain. Dupers had a positive phase angle when entrained, showed signs of scalloping (Morin et al., 1977), and reduced locomotor activity. ***B***, Latency to re-entrain locomotor activity over the course of the experiment. Successive delays (D1, D2…D4) and advances (A1, A2…A4) were alternated (**p < 0.001, ***p < 0.0001, ****p < 0.00001, *t*-test). ***C***, Duper hamsters consistently re-entrained much more quickly than wild types to all phase shifts (combined data from ***B***). ***D***, Mean latencies to re-entrain for each shift type, shown for each genotype. Duper hamsters show no differences in latency to reentrain to delays versus advances. WT hamsters re-entrained more rapidly to delays than advances. Results are shown as the mean ± SEM. As seen in Extended Data Figure 2-1, the mean duration of the active phase (α) was greater in both shifted and unshifted wild-type hamsters than in the corresponding groups of duper mutant hamsters over the course of the experiment (*p* < 0.05), but the mean number of wheel revolutions did not differ between genotypes in either shifted or unshifted hamsters.

In agreement with previous observations in male hamsters (Sisson et al., 2022), duper mutant females re-entrained activity rhythms more rapidly than did wild types after 8 h shifts of the LD cycle. For both delays and advances, dupers re-entrained more than fourfold more rapidly than WTs (for advances, 2.28 ± 0.17 vs 10.76 ± 1.75 d; for delays, 2.65 ± 0.52 vs 14.08 ± 0.78 d, mean ± SEM; *p *<* *0.05; Fig. 2*B*,*C*). Wild-type hamsters re-entrained more quickly to advances than to delays (*p *<* *0.05; Fig. 2*D*). Dupers showed no difference in rates of re-entrainment of phase advances versus delays (*p *>* *0.05; Fig. 2*D*). We found no evidence that the latency to re-entrain changed with repeated shifts: hamsters showed a similar rate of behavioral response to the first and the last advance and delay of the LD cycle. Although activity rhythms were disrupted by 8 h advance and delay phase shifts, daily vaginal smears indicated that with very few exceptions estrous cycles were not interrupted in either genotype (data not shown). In contrast to observations in mice (Pilorz, et al., 2020), the latency to re-entrain was unaffected in either genotype by the day of the estrous cycle on which hamsters were subjected to 8 h advances or delays.

10.1523/ENEURO.0359-22.2023.f2-1 Figure 2-1 The duper mutation reduces α but does not alter the number of wheel revolutions in shifted and unshifted animals. ***A***, The time elapsed between onset and offset of activity is significantly greater in WT than in duper hamsters under both shift conditions (*p < 0.05). ***B***, The average number of wheel revolutions did not differ between dupers and WTs under either shift condition. Download Figure 2-1, EPS file.

The duration of activity (α) was greater in wild-type hamsters than in duper hamsters. The genotype effect on α was more striking in the shifted condition (Extended Data Fig. 2-1*A*). In contrast, the amount of activity (number of wheel revolutions) was similar across the four combinations of genotype and shift condition (Extended Data Fig. 2-1*B*).

### Effect of shifts on neurogenesis in the subgranular zone

**Figure 3.**
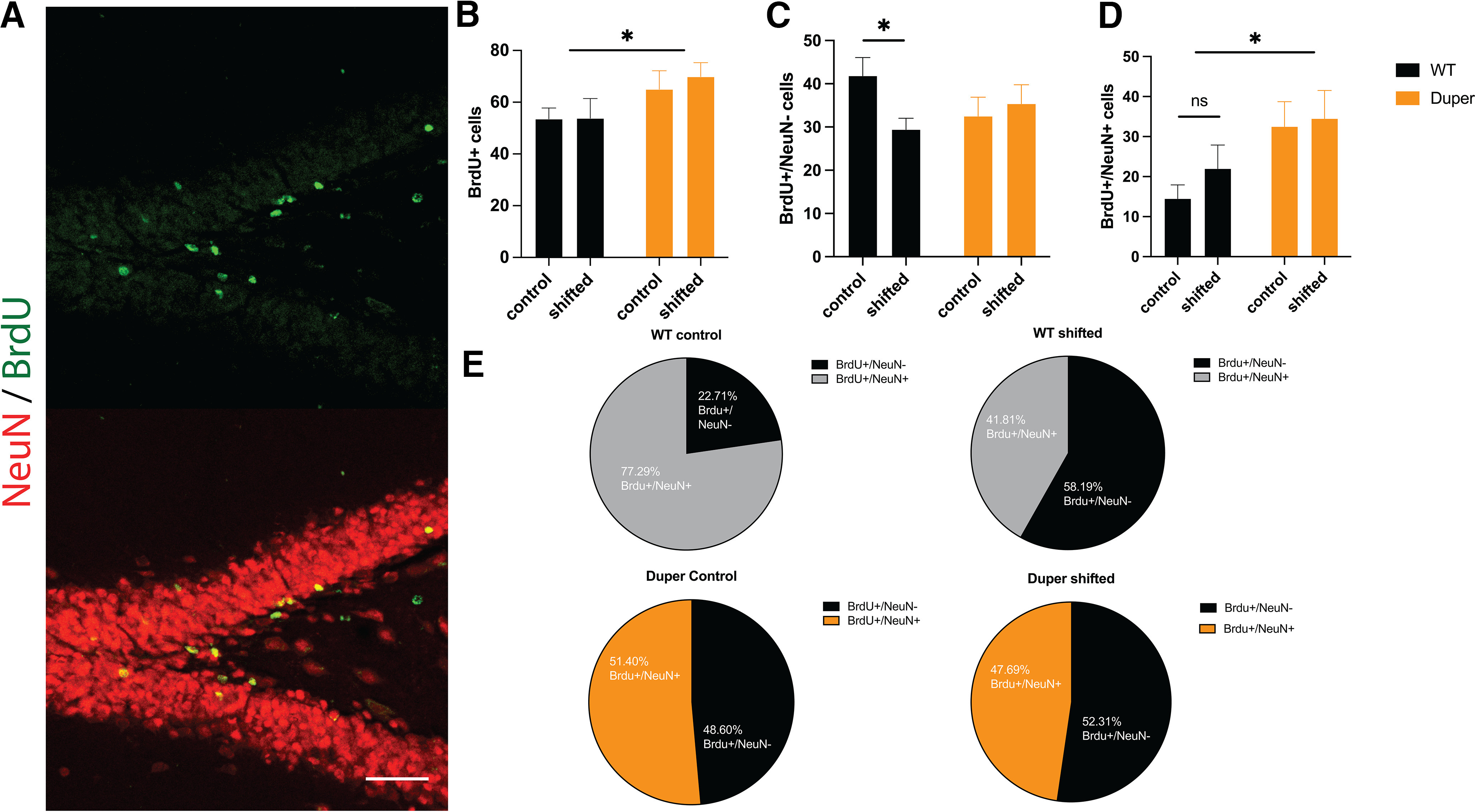
Phase shifts and the duper mutation regulate cell division and neurogenesis in the subgranular zone of the dentate gyrus. ***A***, Representative micrograph showing BrdU (green, top), and BrdU/NeuN (bottom) stained cells in the hamster dentate gyrus. ***B***, Total BrdU^+^ cells were unaffected by circadian disruption in either genotype, but dupers had more BrdU^+^ cells than WTs (*p < 0.05). Results are shown as the mean ± SEM. ***C***, Phase shifts reduced the number BrdU^+^/NeuN^–^ cells in the dentate gyrus in WTs (*p < 0.05) but had no effect on duper mutants. ***D***, Duper mutants showed more newborn neurons (BrdU^+^/NeuN^+^) than WTs (*p < 0.05), but the effect of shifts was not statistically significant in either genotype. ***E***, Pie charts representing the effects of genotype and shifts represented as percentages (%BrdU^+^/NeuN^–^ cells and %BrdU^+^/NeuN^+^ cells). Repeated phase shifts changed the proportion of newborn cells that were NeuN-IR in wild-type (*p* < 0.03) but had no such effect in dupers. Means were compared by two-way ANOVA, with genotype and phase shifts as main factors. As seen in Extended Data Figure 3-1, phase shifts and genotype had no significant effect on BrdU or NeuN staining in the SVZ at the 64 d survival point.

Cell birth was assessed in shifted and unshifted duper and wild-type hamsters. The number of BrdU^+^ cells was quantified in the SGZ of the dentate gyrus of hamsters in each condition (Fig. 3*A*). Shifts had no statistically significant effect in either duper or WT hamsters (*F*_(1,25)_ = 0.06, *p* = 0.81; Fig. 3*B*). However, duper animals (control and shifted combined) had more BrdU^+^ cells than wild types (control and shifted; *F*_(1,25)_ = 4.90; *p* = 0.036; Fig. 3*B*).

10.1523/ENEURO.0359-22.2023.f3-1 Figure 3-1 Neither phase shifts nor genotype affected the number of newborn cells in the subventricular zone at the 64 d survival interval. ***A***, Phase shifts did not affect the number of BrdU- IR cells in either genotype, and the number of BrdU^+^ cells was similar in WT and duper hamsters. ***B***, Schematic illustration of coronal section of a rodent brain showing the SVZ at the origin of the rostral migratory stream. ***C***, Representative micrograph of right ventricle of a coronal section corresponding to ***B***, showing BrdU^+^ cells (green), NeuN^+^ cells (red), and DAPI counterstain (blue). Download Figure 3-1, EPS file.

NeuN was used to identify the phenotypic fate of the BrdU^+^ cells. BrdU^+^/NeuN^–^ cells are considered to be newborn non-neural stem cells (Obernier and Alvarez-Buylla, 2019; Fig. 3*A*). In wild-type hamsters, chronic phase shifts reduced the number of BrdU^+^/NeuN^–^ cells (*t*_(11)_ = 2.68 *p *=* *0.032; Fig. 3*C*). In contrast, repeated phase shifts in the duper mutants had no significant effect on the number of BrdU^+^/NeuN^–^ cells (*t*_(14)_ = 0.461, *p *=* *0.65; Fig. 3*C*).

BrdU^+^ cells colabeled with NeuN are adult-born cells that have differentiated into neurons over the course of the 64 d since the injection. In agreement with evaluation of the total number of cells, phase shifts had a genotype-specific effect on the percentage of BrdU^+^ cells that were colabeled with NeuN (*t*_(11)_ = 2.49, *p* = 0.03; Fig. 3*D*). Similar to the BrdU^+^ data, duper mutants had higher numbers of adult-born neurons (BrdU^+^/NeuN^+^ cells overall) than did wild types (*F*_(1,25)_ = 5.74, *p* = 0.02; Fig. 3*D*). This was also reflected in the percentage of BrdU^+^ cells that were colabeled for NeuN (*F*_(1,25)_ = 6.60, *p* = 0.016; Fig. 3*E*).

In wild types, adult neurogenesis was similar in shifted animals and controls (*t*_(11)_ = 1.478, *p* = 0.16; Fig. 3*E*). However, adult neurogenesis increased in shifted wild types compared with controls when assessed as a percentage of BrdU^+^/NeuN^+^ cells (*t*_(11)_ = 2.49, *p* = 0.03; Fig. 3*E*). No such effects of phase shifts occurred in dupers: the numbers of BrdU^+^/NeuN^+^ cells, and the percentage of BrdU^+^ cells that expressed NeuN, was similar in shifted and unshifted duper hamsters (Fig. 3*E*: *t*_(14)_ = 0.213, *p* = 0.83; and Fig. 3*E*: *t*_(14)_ = 0.099, *p *=* *0.92), respectively.

### Effect of shifts on adult neurogenesis in the subventricular zone

Neurogenesis takes place not only in the SGZ, but also in the SVZ of adult hamsters (Huang et al., 1998). We found few BrdU^+^/NeuN^+^ cells in the SVZ at the time of killing (64 d after injection), and genotype did not affect their number (*F*_(1,24)_ = 1.044, *p *=* *0.31; Extended Data Fig. 3-1). There was no significant effect of phase shifts on the number of BrdU^+^ cells in the SVZ of either genotype (*F*_(1,24)_ = 1.426, *p *=* *0.244; Extended Data Fig. 3-1). Cells that divided in this region likely joined the rostral migratory stream within the 64 d interval between BrdU injection and the time of killing.

### Cell proliferation in the SGZ of shifted and unshifted hamsters

**Figure 4.**
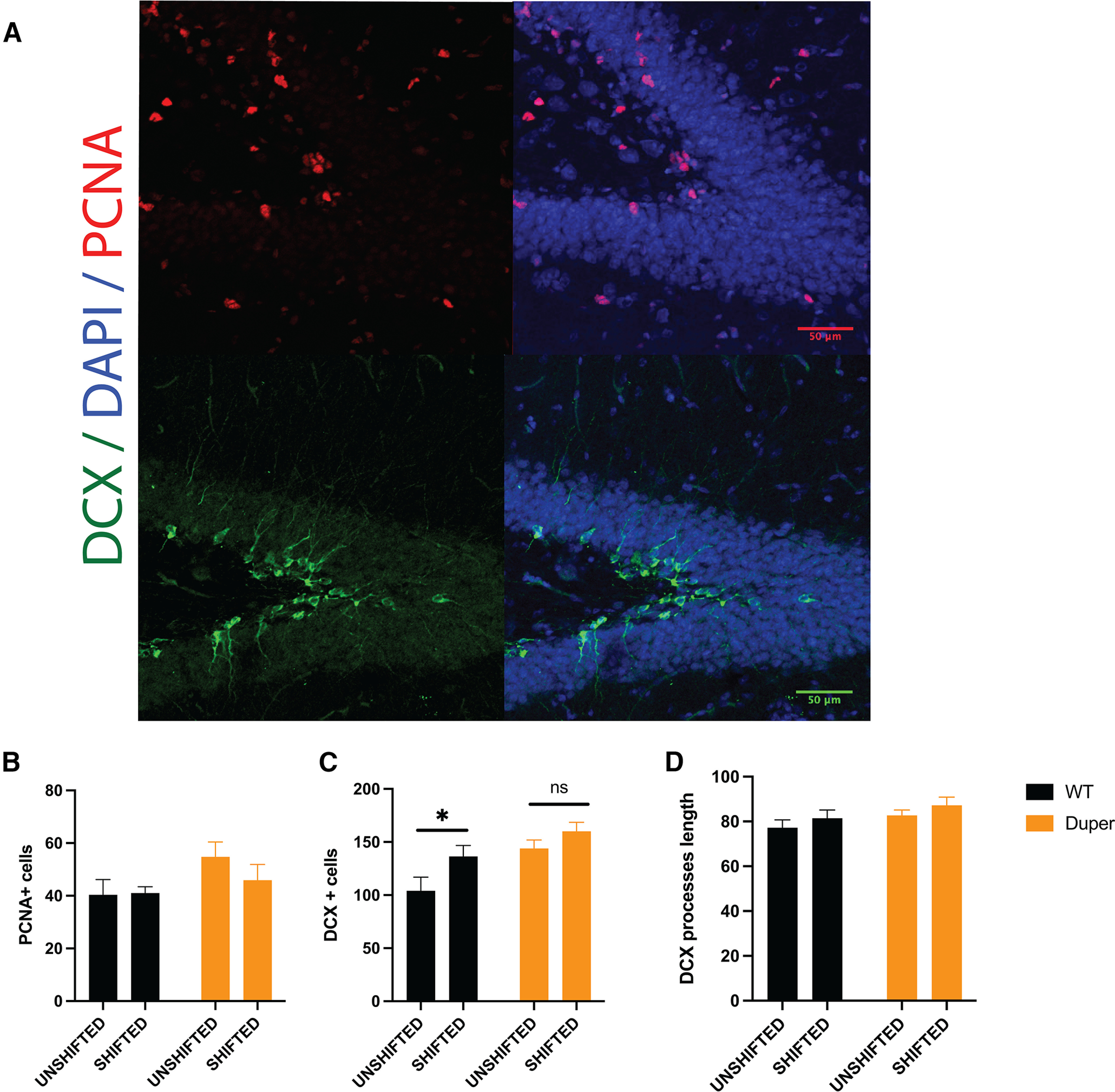
Cell proliferation in the SGZ of shifted and unshifted hamsters. ***A***, Representative images showing PCNA (red, top left) with DAPI counterstain (top right), and DCX (green, bottom left) with DAPI counterstain (bottom, right). ***B***, Neither genotype nor repeated shifts altered the number of PCNA^+^ cells in the subgranular zone, although dupers tended to have more PCNA^+^ cells than WTs (*p* = 0.09). ***C***, Phase shifts significantly increased DCX-IR cell number in SGZ of WTs but were without significant effect (ns) in dupers. *p < 0.05. ***D***, DCX process length was not affected by genotype or phase shifts.

Quantification of BrdU 64 d after injection necessarily indicates a combination of effects on cell birth and survival of genetic and environmental manipulations. Furthermore, the effects of these treatments after eight shifts (131 d) may differ from those that had occurred within 64d, by the time of BrdU administration just before the fifth shift. To assess cell division at the time of killing (after four advance and four delay shifts over the course of the full experiment), we stained for PCNA. The results indicated no statistically significant effect of genotype or repeated shifts on cell division rates at the time of killing (*p* = 0.09; Fig. 4*A*,*B*).

The number of BrdU^+^/NeuN^+^ cells found at killing reflects the number of neural stem cells (NSCs) that were in S phase at 64 d, differentiated into neurons, and survived until killing 9 weeks later. This may not fully reflect effects of the series of phase shifts of the LD cycle on early stages of neuronal differentiation. Thus, we assessed doublecortin expression at the time of killing. DCX-IR was higher in duper hamsters than in WTs (*p* < 0.02) but was not significantly altered by repeated phase shifts (Fig. 4*C*). The length of DCX-IR processes in the hippocampus did not differ with genotype or between shifted and unshifted hamsters (Fig. 4*D*).

## Discussion

The birth and fate of cells in the hippocampus was altered by repeatedly alternating 8 h advance and delay phase shifts. Specifically, circadian disruption reduced the number of adult-born non-neuronal cells in the subgranular zone of the dentate gyrus of wild-type hamsters. Conversely, phase shifts increased the proportion of cells that were born in the SGZ of wild types and differentiated into neurons over the course of the ensuing 9 weeks. Neither of these effects was evident in duper hamsters, a *Cry1*-null mutant strain. Although duper and wild-type hamsters experienced the same manipulations of the LD schedule, the mutants re-entrained far more rapidly. Thus, our results support the hypothesis that sustained internal desynchronization rather than phase shifts per se is a critical component of the deleterious effects of chronic circadian disruption. This finding may be significant for our understanding of the adverse health consequences of shiftwork and jet lag.

Our findings are consistent with previous work indicating an effect of circadian disruption on neurogenesis in hamsters, rats, and mice (Gibson et al., 2010; Kott, 2012; Iggena, 2017; Horsey et al., 2020). Earlier studies used frequent injections of BrdU throughout the experiment (Gibson et al., 2010) or biomarkers of immature neurons rather than quantification of cell birth to assess neurogenesis (Kott et al., 2012; Horsey et al., 2020). Our approach differs in that it quantifies a population of cells tagged acutely during the course of repeated shifts and allowed to mature and differentiate. The comparison of duper hamsters with wild-type hamsters indicates that the disruption of the TTFL, internal desynchrony, or both are critical to adverse effects of phase shifts.

We challenged hamsters with alternating phase advances and delays at 16 d intervals. This allowed us to vary the duration of internal desynchrony while conserving the number and extent of shifts of the LD cycle. Our intention was to design a realistic model of shift work and jet lag, in which humans remain on a particular schedule for many days and re-entrain before reverting to a previous regimen. Such a design is surprisingly uncommon in the literature. Researchers often opt for frequent shifts, which maximizes disruption and does not allow for circadian re-entrainment. This is often done in the interests of obtaining a significant effect but comes at the expense of interpretability and relevance to health. Results of such manipulations should be interpreted carefully. Our design allowed wild-type controls to nearly complete their re-entrainment before each successive phase shift. In contrast to dupers, which experience only a few transient cycles every 16 d, wild-type hamsters re-entrain locomotor activity only after considerable delay. To the extent that behavioral activity reflects the status of the circadian system, it is likely that the wild-type hamsters experienced protracted internal misalignment. It is possible that the difference between wild types and dupers would be reversed if more frequent shifts were imposed, as circadian phase changes less in the first few days after an 8 h advance or delay in the controls than the mutants. The quantitative relationship between the amplitude and frequency of phase shifts and their effect on neurogenesis, as well as other health-related functions, remains to be established.

Our results confirm and extend previous findings using male hamsters in which duper mutants re-entrained much more rapidly than wild types upon 8 h advances or delays of the LD cycle (Sisson et al., 2022). Contrary to previous reports (Reddy et al., 2002; Gibson et al., 2010; Kott et al., 2012), we observed that wild-type animals re-entrained more rapidly to phase advances than delays. Our design did not allow us to determine whether advance or delay shifts differed in their effects on hippocampal neurogenesis and differentiation. Kott et al. (2012) reported that weekly 6 h phase advances reduced the number of immature neurons in the SGZ of rats, but phase delays had little effect. Horsey et al. (2020) also reported weekly phase advances to be particularly effective in reducing the number of immature neurons, and observed changes in dendritic arborization that were correlated with behavioral effects. We did not observe changes in arborization of DCX-IR cells in either wild-type or mutant hamsters (Fig. 4*D*), or examine changes in learning or emotional measures. Studies of the behavioral consequences of wild-type and mutant hamsters subjected to this paradigm are warranted (Fujioka et al., 2011; Toda et al., 2019).

We quantified BrdU-labeled cells that were in S phase at the midpoint of the 8 week experiment and survived over the ensuing 64 d, during which a proportion acquired neuronal phenotype. NSCs generated in adulthood may either become neurons, differentiate into another cell type, or retain stem cell characteristics (Pilz et al., 2018). We find that circadian disruption determines the proportion of NSCs that meets each of these fates in the SGZ. Contrary to our expectations, the percentage of BrdU^+^/NeuN^+^ cells at the time of killing was greater in shifted than control wild types. This effect of phase shifts was not present in the dupers. Furthermore, dupers had more BrdU^+^/NeuN^+^ cells at the time of killing. These effects might reflect elevated levels of adult neurogenesis. This interpretation is consistent with our finding that DCX labeling was higher in dupers. Alternatively, equivalent proportions of newborn cells may initiate neuronal differentiation, but survival may be higher in dupers than in wild-type hamsters, or in shifted than in control wild types. The interval after division at which NSCs reach a choice point that determines which pathway they will enter may be altered by circadian disruption. This may account for the effect of the duper mutation on the proportion of BrdU-IR cells that express NeuN by the time of killing. In mice, circadian rhythmicity is not apparent in intermediate progenitor cells but reappears in neuroblasts (Bouchard-Cannon et al., 2013). Duper mutants may regain the alignment of circadian oscillators more quickly than wild types after a phase shift, and may do so before such a choice point is reached. Thus, phase shifts and genotypes may exert their influence days or weeks after cell division.

The duper allele is a single base deletion leading to a frame shift and stop codon in exon 4 of *Cry 1* (Lee et al., 2022). Thus, our results suggest a role of CRY1, a powerful repressor of the function of BMAL1:CLOCK heterodimers to activate transcription, in the regulation of cell division, differentiation, and survival in the adult hippocampus. Like the products of *Bmal1*, *Clock*, and *Per* genes, CRY1 may influence adult neurogenesis not only through transcriptional regulation within the core circadian loop, but also by influences on the expression of clock-controlled genes, including those that regulate cell division and differentiation (Koike et al., 2012). Furthermore, the effects of circadian disruption may reflect malfunction of the cell-autonomous clocks of the stem cells themselves or of other cell types. Differences between dupers and wild types in the phase relationship of intracellular circadian oscillators, or between cell types within the niche, may result in changes in the proportion of newborn cells that enter the neuronal pathway versus the non-neuronal pathway. Within NSCs, the expression of checkpoint signals and cell-signaling molecules that regulate division and differentiation may be under circadian control (Gréchez-Cassiau et al., 2008; Gonçalves et al., 2016; Draijer et al., 2019; Ali and von Gall, 2022; Mayerl et al., 2022).

Metabolic functions of *Cry1* may also contribute to effects of the duper mutation on adult neurogenesis (Jordan et al., 2017). *Cry1* deficiency increases the proliferation of mouse embryonic fibroblasts through a cell-autonomous mechanism that depends on *Bmal1*, but appears to be dissociable from circadian function (Destici et al., 2011). CRY1 is a negative regulator of the hypoxia-signaling pathway HIF-1ɑ, which modulates cell division in hippocampal progenitor cells (Dimova et al., 2019). The deficiency of *Cry1* in duper mutants may thus cause an increase in cell division in the SGZ through disinhibition of this member of the bHLH-PAS (Per-ARNT-SIM) family. Such an effect may mask the deleterious effects of circadian disruption through frequent phase shifts. Experiments in adult mice and rats indicate that the opportunity for exercise, resulting from providing a running wheel, *increases* adult neurogenesis (van Praag et al., 1999). We found that the duration of the α phase was *decreased* in duper hamsters, while the total number of wheel revolutions did not differ between genotypes (Extended Data Fig. 2-1). Although differences between shifted and control groups in running activity may influence metabolic demands, increases in neurogenesis in duper mutants seem unlikely to be attributable to changes in energy expenditure.

Circadian control of cell division is well established in a variety of cell types (Matsuo, 2003; Brown, 2014), and NSC division in hippocampus peaks during the light phase in Syrian hamsters and mice (Smith et al., 2010; Bouchard-Cannon et al., 2013). Additional experiments with short-interval BrdU survival are needed to assess the effect of the duper mutation on cell division in the hamster SGZ. Not only the increase in BrdU-IR cells but also changes in DCX-IR in duper mutants may result from disinhibition of the positive limb of the TTFL. Our findings on the effects of the duper mutation are consistent with other evidence that the TTFL regulates NSC proliferation and differentiation. *Bmal1* and *Per2* may promote NSC proliferation and differentiation, but also reduce the survival of neurons born in adulthood (Borgs et al., 2009; Bouchard-Cannon et al., 2013; Rakai et al., 2014; Schnell et al., 2014; Malik et al., 2015). Disruption of oscillations of *Per2* and *Bmal1* in the quiescent hippocampal stem cells may disrupt cell cycle entry and exit (Bouchard-Cannon et al., 2013). This is an important form of regulation since uncontrolled cell cycle entrance and exit could rapidly deplete the NSC population. In the absence of CRY1, dupers may experience an increase in NSC proliferation, differentiation, and/or survival that masks, opposes, or overrides the effects of phase shifts. In the absence of phase shifts, a higher proportion of NSCs in the dentate gyrus differentiated into neurons in duper hamsters than in their wild-type counterparts. This appears to contrast with findings that *Cry1*-deficient neurospheres prepared from mouse DG show reduced proliferation (Malik et al., 2015).

The neural pathways by which shifts of the light/dark cycle influence adult neurogenesis remain to be determined. *Cry1* is widely expressed in mouse retina, and *Cry1*-deficient animals show changes in the electroretinogram (Wong et al., 2018). Manipulations of the LD schedule such as those used in the present experiment inevitably trigger not only circadian re-entrainment but also masking, but changes in neurogenesis are attributable to the former (Duy and Hattar, 2017). Entrainment is mediated by light input to the master hypothalamic pacemaker in the suprachiasmatic nucleus (Welsh et al., 2010). Suprachiasmatic nucleus (SCN) efferents (most likely through a septal relay) and/or humoral outputs may mediate circadian control of functions including cell division, rhythmicity, survival, and differentiation in the hippocampus (Fischette et al., 1981; Silver et al., 1996; Fernandez et al., 2014; Kress et al., 2018). The hypothalamic pacemaker may ultimately govern the phase of expression and temporal coincidence of cell cycle regulators. It may be useful to determine whether direct manipulation of the SCN pacemaker, in the absence of changes of TTFL function elsewhere in the brain, can alter the course of cell division and differentiation in neurogenic regions. Light may also influence hippocampal function by a direct pathway that does not involve the circadian system (LeGates et al., 2014). Effects of this pathway on neurogenesis remain to be established, however, so that the possibility that such inputs mediate effects of duper mutation on cell birth and survival remains speculative.

In contrast to the SGZ, we found no difference between dupers and wild types in the number of BrdU^+^ cells in the SVZ. Studies have failed to find circadian rhythms of cell division in the SVZ (Tamai et al., 2008; Draijer et al., 2019). However, the examination of the SVZ 9 weeks after BrdU injection is insufficient to evaluate the effects of *Cry1* deficiency and circadian disruption on adult neurogenesis in this region. Investigation of the effects of the duper mutation and phase shifts on cells that originate in the hamster SVZ and migrate to the olfactory bulb through the rostral migratory stream are warranted (Huang et al., 1998).

In conclusion, our findings indicate that circadian disruption regulates neurogenesis, differentiation, and cell survival in the adult hippocampus. These effects are attributable to misalignment rather than phase shifts per se *Cry1*, a core circadian clock gene, may be critical to neurogenesis and responses to environmental signals.

## Synthesis

Reviewing Editor: Christopher Colwell, UCLA School of Medicine Decisions are customarily a result of the Reviewing Editor and the peer reviewers coming together and discussing their recommendations until a consensus is reached. When revisions are invited, a fact-based synthesis statement explaining their decision and outlining what is needed to prepare a revision will be listed below. The following reviewer(s) agreed to reveal their identity: Norman Ruby.

Overall, the reviewers and the editor are in agreement that this study was done well and results are interesting and informative. The manuscript is well written and graphics clear. The data analysis is fine and the conclusions are mostly justified from the data. Both reviewers had some concerns about the discussion that will need to be addressed in the revision.

Reviewer 1

While the, there is substantial speculation in the Discussion about mechanisms not investigated in the present manuscript. I would suggest reducing speculation or specificity (e.g., speculation about impact on cell signaling, metabolism, etc. without many specific details).

In some cases, it is suggested that WT animals may be differentially affected as Duper animals are protected from sustained internal desynchronization (e.g., lines 13-16 of the Discussion). In others, misalignment is discussed (e.g., line 3, p. 18). I’m assuming that internal desynchronization refers to coordination of optimal phase among central and peripheral oscillators). I’m not sure if misalignment refers to the same or misalignment relative to the LD cycle. Related to these points, it is possible that wheel running is reflective of the phase of the SCN (and possibly other central oscillators) and whether internal desynchronization occurs to the same extent in Duper hamsters is unclear. Clarifying this point would help to improve the impact of the work and provide further support for the conclusions/hypothesis.

Because wheel running can affect cell proliferation/neurogenesis, it would be important to assess whether the extent of voluntary wheel running differs between Duper/WT animals and shifted/static conditions.

Minor comments:

1. There is no reference to Figure 1 in the manuscript. Please reference the Figure in the Methods section.

2. Somewhat minor, but I find it easier to ground myself to graphs when the. control condition is presented on the left. I would suggest moving Duper mice to the right and WT to the left. I also suggest moving controls to the left and shifted to the right.

3. Figure 3 might be simplified by only providing total number of BrdU positive cells, BrdU+/NeuN cells, and % BrdU+/NeuN cells.

4. I may have missed it, but I see no mention of how DCX process length was examined. Is the extent of the process maintained within a single section? Were all DCX cells assessed for process length or only those with complete processes, etc.? Likewise, counts of PCNA and DCX are not described in “Imaging, cell counting and quantification.”

5. Just an observation - in Figure 2, It is interested that morning and evening oscillators appear to separate and reconsolidate in WT, but not Duper animals, following shifts. This may be worth quantifying systematically.

6. The authors might consider results of Due and Hattar, 2017 in the manuscript.

Reviewer 2:

1. The methods section never explains how reentrainment was defined, or how phase was quantified. This is surprising given that entrainment was the point of the paper.

2. Do the authors have any idea why the active phase is so much shorter in duper?

3. I applaud the authors acknowledging that constant phase-shifting of the LD cycle gets results, but at the loss of human relevance, a point seemingly overlooked by everyone studying these models.

4. Phase delays seem to fragment rhythms in wt, but not in duper. The actograms show part of the activity reentraining immediately while the onsets lag behind. By contrast, within the same wt animal, phase advances are consolidated. It seems clear that light is directly disrupting the circadian pacemaker, or perhaps its output. I am surprised that the authors did not run a phase shifting study in DD to test this. The phase advances are more convincing, but to my eyes, it throws a wrench into the idea that delays take longer in wt. One could just as easily say that the offsets show rapid reentrainment to a delay. The onsets lag behind only because light has fragmented alpha. And since light promotes sleep, you may simply have a masking effect of light disrupting alpha by promoting sleep. This interpretation is more consistent with the well-established finding that delays happen much faster than advances.

5. In regards to #2 above, work by Aziz Sancar showed that mice lacking cry1 had reduced per1 induction in the SCN in response to light. This suggests that the SCN of duper is less sensitive to light and so does not fragment its rhythms during phase delays. Given the differences in light-induced rhythm fragmentation, I am concerned that comparing wt and duper solely on reentrainment rates is like comparing apples to oranges-the underlying issue may not be reentrainment per se, but rhythm consolidation. Moreover, if the authors want to make this relevant to humans, then they really need to think more about how rhythm fragmentation plays into the translational relevance of their model. I think the authors need a more thoughtful presentation of the running wheel data in the discussion section.

6. The discussion section needs some editing. Some examples:

Pg. 14, line 21, “The interval...” seems like you are introducing a new idea. Perhaps a paragraph break is needed?

Pg. 15, line 12, “Effects of circadian...” this sentence is introducing a new idea, needs a paragraph break.

Pg. 15, line 20, “Metabolic functions...” also introducing a new idea.

I’ll leave the editing up to the authors, but if you want reader to follow your train of thought, some judicious editing would help.

## Author Response

Synthesis Statement for Author (Required):

Overall, the reviewers and the editor are in agreement that this study was done well and results are interesting and informative. The manuscript is well written and graphics clear. The data analysis is fine and the conclusions are mostly justified from the data. Both reviewers had some concerns about the discussion that will need to be addressed in the revision.

Reviewer 1:

1. While the, there is substantial speculation in the Discussion about mechanisms not investigated in the present manuscript. I would suggest reducing speculation or specificity (e.g., speculation about impact on cell signaling, metabolism, etc. without many specific details).

In response to this comment, we have condensed the discussion to reduce the detailed speculation on cell signaling, etc (p. 16 15 -18). The mechanisms remain to be explored, but we have now refrained from laying out some of the specific possible mediators of the effects of the duper mutation and phase shifts on neurogenesis.

2. In some cases, it is suggested that WT animals may be differentially affected as Duper animals are protected from sustained internal desynchronization (e.g., lines 13-16 of the Discussion). In others, misalignment is discussed (e.g., line 3, p. 18). I’m assuming that internal desynchronization refers to coordination of optimal phase among central and peripheral oscillators). I’m not sure if misalignment refers to the same or misalignment relative to the LD cycle.

We introduce internal misalignment in the abstract (p. 1, l. 5) as “a state in which abnormal phase relationships prevail within and between organs.” The passage in the introduction (p. 3, l. 16-17) emphasizes the role of the circadian program not only to optimize internal phasing but also control phasing with respect to the environment. While both functions are important, we have reserved the use of “misalignment” and “internal desynchrony” to refer to the internal phasing, although we cannot yet specify which events or physiological processes may vary during transients of entrainment or between dupers and wild types.

3. Related to these points, it is possible that wheel running is reflective of the phase of the SCN (and possibly other central oscillators) and whether internal desynchronization occurs to the same extent in Duper hamsters is unclear. Clarifying this point would help to improve the impact of the work and provide further support for the conclusions/hypothesis.

We have revised the passage on p 14 (l. 10-12) of the the ms to clarify that misalignment refers to an altered phase of locomotor activity with respect to the LD cycle. In response to the reviewer’s comment we have expanded the text to spell out more clearly the inference that slow re-entrainment of locomotor rhythms in the wild types is likely to reflect the status of the entire circadian system, and thus indicate a protracted state of internal misalignment.

For the reviewer’s information, we have performed further experiments to evaluate this inference. First, we have examined the time at which the preovulatory LH surge occurs in wt and duper hamsters subjected to an 8h advance. Our results document that the mutants complete re-entrainment of the LH surge within 4 days (one estrous cycle), while the wild types have not yet done so. Second, we have completed an RNA-Seq analysis of the transcriptional changes in the liver of duper vs. wt hamsters subjected to an 8h phase advance. Shifts of transcripts of most of the core clock genes occur much more quickly in duper than in wt, and this is also true of many clock controlled genes. We are complementing this analysis with further RNA-Seq on heart, lung and skeletal muscle, as well as with immunocytochemical analysis of PER1 and PER2 in the SCN. Each of these studies will be the subject of a separate manuscript. We feel that inclusion of these results in the present ms would distract from the main points about neurogenesis. If it is consistent with editorial policy for eNeuro, we can revise the discussion to include a general reference to “unpublished findings” that support the inference that our behavioral data (Fig. 2) reflects the behavior of internal physiological and transcriptional events.

4. Because wheel running can affect cell proliferation/neurogenesis, it would be important to assess whether the extent of voluntary wheel running differs between Duper/WT animals and shifted/static conditions.

We agree with the reviewer’s comment about the possible contribution of effects of the duper mutation on voluntary wheel running and its potential influence on neurogenesis. In response to the reviewer’s comment, we have revised the discussion (p. 17, l. 3-8) to address this possibility.

A previous analysis of alpha in wt and duper, as well as super short and super duper hamsters (PLoS One 7(5):e36119, 2012; doi: 10.1371/journal.pone.0036119) indicates that alpha did not differ significantly between dupers and wild types although dupers generated more wheel revolutions per circadian cycle (see Table 1 and Figure 5, top). For the reviewer’s information, we have done a more extensive analysis of differences between wt, duper, and tau heterozygote female hamsters in wheel running. We find that not only the entrained phase angle (scalloping) but also alpha and the total number of wheel revolutions differ significantly between genotypes, and that these differences vary across the days of the estrous cycle. We feel that this subject is important but complex and we are preparing a separate manuscript to address it in greater depth. To include this analysis in the current ms would in our opinion distract from the main issues concerning Cry1 deficiency, responses to phase shifts, and neurogenesis.

Minor comments:

1. There is no reference to Figure 1 in the manuscript. Please reference the Figure in the Methods section.

In response to the reviewer’s comment, we have added the reference for Fig 1 in the methods section (Pg. 5, line 18).

2. Somewhat minor, but I find it easier to ground myself to graphs when the. control condition is presented on the left. I would suggest moving Duper mice to the right and WT to the left. I also suggest moving controls to the left and shifted to the right.

In response to the reviewer’s comment, we have adjusted the figures to suit more traditional representation of control vs shifted and wt vs mutant.

3. Figure 3 might be simplified by only providing total number of BrdU positive cells, BrdU+/NeuN cells, and % BrdU+/NeuN cells.

We agree with the reviewer that simplification of the data in Fig. 3 would be helpful. Accordingly, we have changed the presentation of the data in Fig. 3E from bar graphs to pie charts as was done in other adult neurogenesis papers (Bouchard-Cannon et al., 2013)

4. I may have missed it, but I see no mention of how DCX process length was examined. Is the extent of the process maintained within a single section? Were all DCX cells assessed for process length or only those with complete processes, etc.? Likewise, counts of PCNA and DCX are not described in “Imaging, cell counting and quantification.”

In response to this comment, we have described in the methods section (p. 8, l. 15-19) that we outlined each DCX-ir process within each stack using the draw feature of Fiji. We quantified DCX process lengths by averaging all processes within each stack. Inevitably, the length of projections that were not entirely within the plane of section were underestimated.

5. Just an observation - in Figure 2, It is interested that morning and evening oscillators appear to separate and reconsolidate in WT, but not Duper animals, following shifts. This may be worth quantifying systematically.

The fragmentation of the locomotor activity in hamsters upon shifts of the LD cycle is complicated by masking, particularly during 8h phase delays. The animals show a combination of re-entrainment of the circadian system and masking: suppression of locomotor activity due to their avoidance of exposure to light. We have examined this in males by combining 8h shifts with transfer of animals to DD after the first 2 shifted cycles (Sisson et al., PNAS 2022; https://doi.org/10.1073/pnas.2121883119). This allows us to better assess the phase of the oscillator. We could not apply this procedure in the current experiment without compromising the LD manipulation whose effect on cell division we studied, although it’s an interesting idea for future experiments in which the acute effects of a shift on neurogenesis might be examined.

6. The authors might consider results of Due and Hattar, 2017 in the manuscript.

The report of Duy and Hattar (2017) is indeed relevant, and pertains to the preceding comment about the potential effects of masking. We interpret the lack of effects of T7 exposure on neurogenesis to indicate that it is the circadian response, not other effects of light, that is most relevant to cell division and differentiation in the hippocampus. In response to the reviewers we have cited this paper in the discussion (p. 17, l. 13).

Reviewer 2:

1. The methods section never explains how reentrainment was defined, or how phase was quantified. This is surprising given that entrainment was the point of the paper.

As previously stated (Sisson et al., 2022), we consider entrainment to be complete when the onsets of locomotor activity achieve a stable slope, parallel to LD transitions, as indicated by the linear regression fit provided by the Actimetrics software. In response to this comment, we have revised the methods section (p. 5, l. 22) to include this definition in the ms.

2. Do the authors have any idea why the active phase is so much shorter in duper?

As indicated in our response to reviewer 1 (see #4 above), previously published data (PLoS One i: 10.1371/journal.pone.0036119) established that duper increases the number of wheel revolutions but had no statistically significant effect on alpha.

3. I applaud the authors acknowledging that constant phase-shifting of the LD cycle gets results, but at the loss of human relevance, a point seemingly overlooked by everyone studying these models.

We appreciate the reviewer’s comment on this matter.

4. Phase delays seem to fragment rhythms in wt, but not in duper. The actograms show part of the activity reentraining immediately while the onsets lag behind. By contrast, within the same wt animal, phase advances are consolidated. It seems clear that light is directly disrupting the circadian pacemaker, or perhaps its output. I am surprised that the authors did not run a phase shifting study in DD to test this. The phase advances are more convincing, but to my eyes, it throws a wrench into the idea that delays take longer in wt. One could just as easily say that the offsets show rapid reentrainment to a delay. The onsets lag behind only because light has fragmented alpha. And since light promotes sleep, you may simply have a masking effect of light disrupting alpha by promoting sleep. This interpretation is more consistent with the well-established finding that delays happen much faster than advances.

This comment is similar to point 5 raised by reviewer #1, and we reiterate that advances are easier to evaluate as activity onsets occurring through the dark phase are not complicated by masking. Thus we disagree that light is disrupting the circadian pacemaker; by definition, masking is a change in activity that is independent of the pacemaker. The evaluation of advance vs. delay latencies is objectively quantified (see above). Also as mentioned in the response to reviewer 1, experiments in which hamsters are transferred to DD on the second cycle after the shift of the LD cycle provide an evaluation of the phase of the pacemaker independent of masking (Sisson et al. PNAS 2022 https://doi.org/10.1073/pnas.2121883119). This analysis addresses the suggestion of the reviewer, and verifies our conclusion that the dupers shift much farther than the wild types in the initial response to the shift, but does not provide a comparison between the latency to complete re-entrainment upon delays vs. Advances as the LD cycle is discontinued.

We agree that a focus on the offset of activity often provides a different picture of the shift than is evident from exclusive attention to onsets. This is particularly true in mice, and leads some investigators to quantify shifts using offsets (see Fig. 1A in Yamaguchi et al., Science 342:85-90; DOI: 10.1126/science.1238599). On the other hand, onsets are typically used in evaluation of period. There is no doubt that the distribution of wheel revolutions across the active phase changes as putative E and M oscillators alter their coupling. We are unaware of statistical methods to quantitatively evaluate coupling and distribution of activity bouts.

The issue of sleep seems to us to be a separate question, though an important one. It would be worthwhile to monitor sleep with polysomnography as animals shift, and to correlate with behavioral rhythms and effects on neurogenesis. Unfortunately, this is beyond the scope of the present study.

5. In regards to #2 above, work by Aziz Sancar showed that mice lacking cry1 had reduced per1 induction in the SCN in response to light. This suggests that the SCN of duper is less sensitive to light and so does not fragment its rhythms during phase delays. Given the differences in light-induced rhythm fragmentation, I am concerned that comparing wt and duper solely on reentrainment rates is like comparing apples to oranges-the underlying issue may not be reentrainment per se, but rhythm consolidation. Moreover, if the authors want to make this relevant to humans, then they really need to think more about how rhythm fragmentation plays into the translational relevance of their model. I think the authors need a more thoughtful presentation of the running wheel data in the discussion section.

The reviewer seems to be referring to the report by Thresher et al. (Science 282:1490-4, 1998), in which Cry2 (not Cry1) expression in the mouse retina was implicated in amplitude of the Per1 response to light. We note that the effect of Cry2 deletion on phase shifts reported by Thresher was not replicated by Spoelstra et al. (J Biol Rhythms 19(6)518-529, 2004; DOI: 10.1177/0748730404268122). However, we note that Cry1 is expressed in mouse retina (Wong et al., FASEB J 32(8):4302-4314 2018; doi: 10.1096/fj.201701165RR) and its deletion in duper may contribute to the transduction of light inputs to the clock.

However, previous data indicate that the intensity of light required to elicit a phase shift is similar in wt and duper hamsters (see Fig. 1 in J Biol Rhythms 29 (2): 97-109 (2014) DOI: 10.1177/0748730413520399). Furthermore, the duper SCN is more responsive than wt to light induction of PER1-ir (as well as FOS-ir; see Manoogian et al., Eur J Neurosci 48:3319-3334, 2018; DOI: 10.1111/ejn.14214). For the reviewer’s information, we are examining Per1 and Per2 expression using immunocytochemical methods in duper and wt hamster SCN. In addition, we are using luminometry to examine regional changes and damping in SCN explants prepared from transgenic Per2:luc wt and duper hamsters. So far we can say that rhythmicity damps more quickly in the duper than the wt SCN, but it is difficult to relate this to rhythm consolidation in vivo. These will help to address the reviewers’ concerns, but are beyond the scope of the present paper.

As described above, we have revised the discussion to discuss the relevance of masking (p. 17, l. 11-14).

6. The discussion section needs some editing. Some examples:

Pg. 14, line 21, “The interval...” seems like you are introducing a new idea. Perhaps a paragraph break is needed?

The idea that changes in the TTFL, such as Cry1 deletion, alters the time course over which the fate of cells born in adulthood, is relevant here and interprets the finding that the duper mutation and LD shifts alter the proportion of BrdU-ir cells that express NeuN. In response to the reviewer’s comment, we have added a sentence to this effect (p. 15, l. 19-22).

Pg. 15, line 12, “Effects of circadian...” this sentence is introducing a new idea, needs a paragraph break.

As an earlier critical comment noted, many possible mechanisms may contribute to the effects of the duper mutation and phase shifts on neurogenesis and we have been admonished not to go overboard in speculation. We feel that the idea that different cell types may be involved, as well as clock-controlled genes beyond those implicated in the TTFL. In response to the reviewer’s comment, we have begun the sentence in question with “Furthermore..” (p. 16. l. 11) but otherwise left the paragraph structure intact.

Pg. 15, line 20, “Metabolic functions...” also introducing a new idea. I’ll leave the editing up to the authors, but if you want reader to follow your train of thought, some judicious editing would help.

We feel that metabolic functions are suitably included in the same paragraph as the comments about checkpoint signals and signaling molecules, as well as with the thoughts about HIF1a and running activity that are also included here. We have used judgement in our editing, but agree that it is challenging to raise multiple ideas in a succinct manner. In our opinion, this challenge is attributable to the rich set of results and intriguing possibilities they raise.

We have made some minor changes to improve grammar and syntax. On instructions from the eNeuro editorial staff that we received in the initial quality check, we have changed the designation of the figure showing negative results on BrdU labeling in the subventricular zone from Supplementary Fig. 1 to Fig. 3-1. This necessitates moving the last paragraph of the results section to follow the description of effects of phase shifts and genotype on BrdU and NeuN labeling in the dentate gyrus (p. 11 l. 19- p. 12 l.2).
